# Changes in colorectal cancer knowledge and screening intention among Ohio African American and Appalachian participants: The screen to save initiative

**DOI:** 10.1007/s10552-021-01462-w

**Published:** 2021-06-24

**Authors:** Andrew S. Boutsicaris, James L. Fisher, Darrell M. Gray, Toyin Adeyanju, Jacquelin S. Holland, Electra D. Paskett

**Affiliations:** 1 College of Medicine, The Ohio State University, Columbus, OH 43201 USA; 2 Arthur G. James Cancer Hospital and Richard J. Solove Research Institute, 1590 N. High St. Suite 525, Columbus, OH 43210 USA; 3 Comprehensive Cancer Center, The Ohio State University, Columbus, OH 43201 USA; 4 Division of Cancer Prevention and Control, Department of Internal Medicine, College of Medicine, The Ohio State University, Columbus, OH 43210 USA; 5 Division of Epidemiology, College of Public Health, The Ohio State University, Columbus, OH 43210 USA

**Keywords:** Colorectal cancer, Screening, Inflatable Colon, Colonoscopy, Disparities

## Abstract

African Americans and Appalachians experience greater incidence and mortality rates of colorectal cancer due to factors, such as reduced prevalence of screening. An educational session (the Screen to Save Initiative) was conducted to increase intent to screen for colorectal cancer among African Americans and Appalachians in Ohio. Using a community-based approach, from April to September 2017, 85 eligible participants were recruited in Franklin County and Appalachia Ohio. Participants completed a knowledge assessment on colorectal cancer before and after participating in either an educational PowerPoint session or a guided tour through an Inflatable Colon. Logistic regression models were used to determine what factors were associated with changes in colorectal cancer knowledge and intent to screen for colorectal cancer. The majority (71.79%) of participants gained knowledge about colorectal cancer after the intervention. Multivariate results showed that race (OR = 0.30; 95% CI: 0.11–0.80 for African Americans versus White participants) and intervention type (OR = 5.97; 95% CI: 1.94–18.43 for PowerPoint versus Inflatable Colon) were associated with a change in knowledge. The association between education and intent to screen was marginally statistically significant (OR = 0.42; 95% CI: 0.16–1.13 for college graduate versus not a college graduate). A change in colorectal cancer knowledge was not associated with intent to screen. Future educational interventions should be modified to increase intent to screen and screening for colorectal cancer. Further research with these modified interventions should aim to reduce disparities in CRC among underserved populations while listening to the voices of the communities.

## Introduction

For both men and women combined, colorectal cancer (CRC) is the second leading cause of cancer mortality in the United States [1]. However, it is preventable with screening and largely curable when detected at an early stage. The five-year relative survival probability for CRC diagnosed at a localized stage is 90% [4]. Several screening methods (colonoscopy, flexible sigmoidoscopy, and fecal occult blood testing (FOBT), and fecal immunochemical testing (FIT)) offer CRC prevention and/or detection [5].

The CRC burden is unevenly distributed along factors, such as race, socioeconomic status (SES), and geography [6–9]. In particular, African Americans (AA) and those residing in the Appalachian (APP) region of the United States, a 420 county region spanning 13 states along the Appalachian Mountains, experience disproportionately higher CRC burdens [10, 11]. The average annual (2014–2018) age-adjusted CRC incidence rate in Ohio is similar among AA and White Ohioans (40.7 per 100,000 White Ohioans, 39.8 per 100,000 AA Ohioans) [12]; however, the mortality rate is 24.6% higher among AA (14.7 versus 11.8 per 100,000) [12]. In 2018, the estimated percentage of AA Ohioans who met the United States Preventive Services Task Force (USPSTF) CRC screening recommendation was slightly lower (66.5%) than the percentage of whites (67.6%) [13]. In the United States, the percentage of CRC cases diagnosed at the distant stage is greater among AA as compared to Whites (26% versus 22%) and the five-year relative survival probability is lower among AA (58.8% versus 65.0%) [14].

A multitude of factors contribute to the differential risk of CRC among AA. For example, there are biological elements, such as single nucleotide polymorphisms that may partially explain the differences in CRC incidence rates [15, 16]. However, social determinants of health also influence the conditions (i.e., environment and access to health care) and health behaviors (i.e., lifestyle and CRC screening behaviors) that contribute to CRC risk [17–20]. Therefore, it is important to recognize the multilevel influences on health from genetics to society, and to use a holistic approach when addressing the issue [21, 22]. The culmination of these multilevel factors result in cancer diagnoses at later stages and lower five-year relative survival for AA compared to non-Hispanic Whites [24]. The increased CRC mortality rates for AA as compared to non-Hispanic Whites exists even within the same SES groups as well [25].

The APP populations experience a high risk of CRC as well. CRC incidence and mortality rates among APP men and women in Ohio are greater than those of non-APP residents of Ohio [11, 26]. The average annual (2014–2018) age-adjusted CRC incidence rate among APP residents in Ohio is 6.9% greater than that among non-APP Ohioans (43.6 per 100,000 APP Ohioans, 40.8 per 100,000 non-APP Ohioans) [12]; the mortality rate is 7.6 higher among APP Ohioans (12.7 versus 11.8 per 100,000) [12]. Scioto County, an APP county in Ohio and an intervention site for the current study, was one of the counties identified as a geographic hotspot for CRC mortality and one of the areas that would benefit from improved screening [8]. In Appalachia, there are a number of barriers for CRC screening faced by patients (e.g., insufficient or incorrect knowledge about CRC screening) and health care providers (e.g., limited time for patient encounters). [27, 28]. The combination of these barriers at the level of the patient and physician generates low CRC screening prevalence, with only 30.1% of APP women being within recommended guidelines for CRC screening [29].

Theoretical models can be used to predict behavior. For example, the theory of planned behavior may provide a framework for understanding this effort. The theory is centered on individual intention to perform a behavior, which is determined by the independent elements of attitude towards the behavior, subjective norms, and perceived behavioral control. The theory of planned behavior model has practical applications for increasing cancer screening among people with insufficient or limited knowledge [33, 34]; increasing awareness and education about CRC has been demonstrated to increase intent to screen for CRC [35, 36]. Intent to screen is important since it is associated with obtaining CRC screening [37, 38].

### Current study

Therefore, an intervention was conducted to increase CRC knowledge to improve CRC screening rates in AA and APP populations. The primary aim was to determine if a change in knowledge of CRC is associated with intent to screen for CRC. The secondary aims were to elucidate what factors were associated with a change in CRC knowledge and intent to screen for CRC.

## Methods

### Study design

The Screen to Save (S2S) initiative was a national effort funded by the Center to Reduce Cancer Health Disparities (CRCHD) and National Cancer Institute (NCI) aimed at enhancing knowledge and promoting healthy behaviors to address health disparities in CRC for at-risk groups [39]. As part of the national effort, investigators at The Ohio State University conducted education sessions from April 17, 2017 to September 30, 2017 to increase CRC knowledge and screening intent for several vulnerable populations across Ohio. The study design was a pre/posttest one sample design with each participant receiving an educational intervention about CRC and CRC screening. This project was approved by the Ohio State University Cancer IRB.

Participants came from five locations in Franklin County, a metropolitan county of over 1.3 million residents with more racial diversity than most other Ohio counties, with 33.2% non-White and 23.8% AA residents, and from two Appalachian counties in Ohio—Jackson County, with approximately 32,000 residents and little racial heterogeneity (3.2% non-White and 0.7% AA), and Scioto County, with approximately 75,000 residents and 5.6% non-White residents and 2.7% AA. The Appalachian region of Ohio includes 32 counties of the Southern and Eastern region. These locations were chosen to target AA and APP, respectively, but participation was open to men and women of all races and ethnicities who were 50–74 years old. To use a diverse array of community outreach efforts, participants were recruited using posted flyers, personal communications, emails through listservs, and church bulletins. Community health educators (CHE) of various backgrounds, including a registered nurse, who were employees of The Ohio State University, conducted educational sessions with participants. The Ohio State University Comprehensive Cancer Center is deeply connected to communities in both Central Ohio (Franklin County) and Appalachia Ohio (Jackson and Scioto Counties) through outreach and education efforts [40–43]. CHEs employed in this study had worked with community members for at least several years and were well-respected by community stakeholders. Interventions in Franklin County took place at Marion Franklin Community Center (two events), Gillie Community Senior Center, The Ohio State University Wexner Medical Center, Dodge Community Center, and Antioch Baptist Church. Interventions in Appalachian counties took place at the New Lexington Library and the Bloom-Vernon School District Health Fair in South Webster, Ohio. Informed consent was obtained from all individual participants included in the study.

In total, surveys (described below) were collected from 104 participants across community centers and local health fairs, but only 85 participants met the age eligibility criterion (50–74 years). All participants received a study information sheet prior to providing verbal consent. Prior to receiving either intervention (described below), participants were given a short survey and assessment. The survey, which was developed by the CRCHD National Outreach Network (NON), obtained demographic information, such as age, gender, race, language, education level, and health insurance coverage. Specific information related to CRC, such as screening and family history were collected as well. Survey questions included those modified from the existing instruments from peer-reviewed literature and those constructed to reflect topics not included in the existing surveys [39]. To validate the survey questions, surveys were pilot tested with the intended target audiences in collaboration with the NON CHE [39]workgroup. The assessment consisted of 14 questions that determined the level of knowledge about basic CRC information, including risk factors and screening. At the conclusion of the intervention session, an identical assessment was given. Additional questions were asked about intention to “talk to a healthcare provider about colorectal cancer screening”, “get screened for colorectal cancer”, “talk about colorectal cancer with family members or friends”, “eat healthier”, and “increase my physical activity” with the questions rated on a 4-point Likert-type scale, as “Strongly Disagree” to “Strongly Agree”.

The type of education session intervention varied based on the site location. One intervention consisted of a PowerPoint presentation for a group led exclusively by a CHE who covered basic knowledge about CRC and screening (offered at the Gillie Community Senior Center, New Lexington Library, Dodge Community Center, and Antioch Baptist Church). This intervention follows a traditional template of using small media as an effective educational tool [44]. The other intervention tested was more nontraditional and used a guided tour through an Inflatable Colon depicting pathology associated with CRC to teach general information about CRC and screening (offered at the Bloom–Vernon School District Health Fair, Marion Franklin Community Center, and The Ohio State University Wexner Medical Center). The Inflatable Colon used was similar to the model utilized in other studies [45–48]. The Inflatable Colon tour guides were CHEs who stressed the same key messages and integrated culturally appropriate messages to their audiences across sites to ensure consistency. The content for both interventions was derived from the National Cancer Institute’s early detection messages [49]. All participants also received an informative handout on CRC and screening at the conclusion of participation.

### Measures

The primary outcomes were change in CRC knowledge and intent to screen for CRC. Change in knowledge (ΔK) was measured by subtracting the participant’s score on the preintervention assessment from his or her score on the postintervention assessment. The domain for ΔK is *i* ϵ ℤ ∩ [− 14, 14], with negative values indicating a reduction in knowledge after the intervention and positive scores representing an increase in knowledge after the intervention. The standardized change in knowledge (S_k_) was calculated for each participant to account for the variability in preintervention scores. The measure is derived from the following:

$$S_{K} \; = \;\frac{{\Delta K}}{{14 - P}}$$

where P is the preintervention score with a domain of *I* ϵ ℤ ∩ [0, 14] and the range for S_k_ is [− 1,0) $\cup$ (0,1]. Therefore, standardized change in knowledge represented the fraction of knowledge gained or lost that was available for each individual to learn. Standardized change in knowledge, rather than change in knowledge, was used in analyses.

Screening intent was determined from the postintervention question assessing likelihood to “get screened for colorectal cancer”. Intent to screen was measured on a 4-point Likert-type scale for each question with answers ranging from 1 to 4. Responses were categorized as “Strongly Agree” if participants recorded a score of 4 and categorized as “Do Not Strongly Agree” if a combined score of 1–3 was measured.

The preintervention survey collected the primary independent variables on demographic information which included: age (50–74 years old), gender (male, female, other), race (American Indian/Alaska Native, Asian, Black/AA, Native Hawaiian or Other Pacific Islander, White), education (8th grade or less, some high school without a diploma, high school diploma or GED, some college without a degree, and college graduate), and insurance (none, covered through work, covered through individual plan, covered through military, covered through Medicare, covered through Medicaid). To allow for sufficient sample sizes, age was categorized as 50–65 or 66–74 years old, gender as either male or female, race as White or AA, and education as either college graduate or not a college graduate. In addition, insurance was categorized as private only, Medicare only, or other.

The preintervention survey asked participants if they have ever been screened for CRC. In addition, they could indicate whether they had an at-home stool test like FOBT/FIT, a colonoscopy, and/or used another method of screening. The date of the most recent screening was obtained for each method as well. Using the CRC screening guidelines set forth by the USPSTF, a participant was categorized as “within guidelines” if he or she had an at-home stool test within the past year, a colonoscopy within the past 10 years, or a flexible sigmoidoscopy within the past 5 years [5]. In contrast, a participant was categorized as “outside guidelines” if they have never been screened for CRC or if the date listed for their most recent screening did not meet the USPSTF criteria. Participants lacking a date associated with their last screening were categorized as outside the guidelines for screening.

In the preintervention survey, participants were asked if they ever had a father, mother, brother, sister, child, or niece/nephew diagnosed with CRC. They were asked for the same information about both their paternal and maternal grandfather, grandmother, aunt, and uncle. An additional section for each relative allowed participants to indicate if the CRC diagnosis occurred before age 50. Any responses marked “Yes” were categorized as having a family history of CRC, while those marked “No” or “Don’t Know” were categorized as not having a family history of CRC.

### Statistical analyses

Descriptive statistics (i.e., proportions) were used to describe demographic and additional characteristics of participants and to compare factors of interest according to standardized change in knowledge and in intent to screen for CRC. Logistic regression was used to obtain odds ratios to examine potential associations between factors of interest and both standardized change in knowledge and intent to screen for CRC. Multivariate logistic regression was used to build two models of factors associated with each outcome separately. Models were built backwards, initially including all potentially important factors of interest and, one by one, removing factors that were not associated with outcomes. Removal of factors stopped when all factors in the final model were at least marginally statistically significant. Then, factors which were removed earlier in the model building process were reconsidered for model inclusion, to be certain that removal from the model had not been based only on order of removal. The p values were 2-sided, with alpha set at 0.05 for hypothesis tests. Statistical analyses were conducted using SAS Software version 9.4.

## Results

### Participant characteristics

Demographic and CRC characteristics of the participants are listed in Table 1. The majority of the participants were 50–65 years old, female, AA, did not graduate college, and insured in other ways besides either private insurance only or Medicare only. For their medical history, most participants were within screening guidelines for CRC and did not have a family history of CRC. Almost two-thirds (66%) received the Inflatable Colon intervention, while the remainder (34%) attended a PowerPoint session. There were no statistically significant differences in demographic factors between those who received the Inflatable Colon and those who attended the PowerPoint session.

**Table 1:** Demographic and CRC characteristics for participants (*n* = 85) in the Screen to Save Initiative, April 2017 to September 2017

Characteristic		N (%)
Age^*^	50–65	49 (59.04)
	66–74	34 (40.96)
Gender	Female	59 (69.41)
	Male	26 (30.59)
Race	White	28 (32.94)
	AA	57 (67.06)
Education^*^	Not a college graduate	47 (56.63)
	College graduate	36 (43.37)
Insurance	Private only	24 (28.24)
	Medicare only	23 (27.06)
	Other	38 (44.71)
Screening history for CRC	Outside guidelines	39 (45.88)
	Within guidelines	46 (54.12)
Family history of CRC	No	60 (70.59)
	Yes	25 (29.41)
Intervention	Inflatable colon	56 (65.88)
	PowerPoint	29 (34.12)

*Information about age and education was missing for two participants

### Assessment scores and gains in CRC knowledge

There were 7 participants with a preintervention score of 14; these participants were removed for any analysis involving S_k_ to avoid an indeterminate result. Furthermore, these participants all scored a 14 on the postintervention assessment, so the intervention had neither a beneficial nor deleterious effect on them.

Table 2 shows median knowledge scores before and after the intervention, as well as the percentage of participants with improved knowledge scores. The total median preintervention score was 11.00. Those with the lowest preintervention scores were outside the screening guidelines, while those with the highest preintervention scores were White and college graduates. After the intervention, the overall median score was 12.00. Those with the lowest postintervention scores were participants who did not graduate college and those with insurance through Medicare only; those with the highest postintervention scores were White, insured through private insurance only, had other forms of insurance, and received the PowerPoint presentation. Examining change in knowledge, scores increased for 71.79% of participants. Participants with the lowest percentages of change in knowledge were insured through Medicare only and participants with the highest percentages were insured through private insurance only. Finally, percentages of participants having a standardized change in knowledge greater than or equal to the median value (0.35) were calculated. Those with the lowest percentages of a standardized change of at least 0.35 were those insured through Medicare only and those with the highest percentages were those who received the PowerPoint presentation.

**Table 2:** Assessment scores and gain in knowledge for participants in the Screen to Save Initiative, April 2017 to September 2017

Characteristic		Preintervention score median	Postintervention score median	ΔK > 0 (%)	S_k_ ≥ 0.35 (%)
Age	50–65^*^	11.00	12.00	68.89	46.67
	66–74	11.00	12.00	77.42	58.06
Gender	Female^*^	11.00	12.00	73.08	50.00
	Male	11.00	12.00	69.23	53.85
Race	White^*^	12.00	13.00	74.07	70.37
	AA	11.00	12.00	70.59	41.48
Education	Not a college graduate^*^	11.00	11.00	69.57	47.83
	College graduate	12.00	12.00	73.33	53.33
Insurance	Private only^*^	11.00	13.00	90.91	59.09
	Medicare only	11.00	11.00	42.11	31.58
	Other	11.00	13.00	75.68	56.76
Screening History for CRC	Outside guidelines^*^	10.00	12.00	72.97	45.95
	Within guidelines	11.00	12.00	70.73	56.10
Family History of CRC	No^*^	11.00	12.00	74.07	50.00
	Yes	10.50	12.00	66.67	54.17
Intervention	Inflatable colon^*^	11.00	12.00	64.81	38.89
	PowerPoint	11.00	13.00	87.50	79.17
Total		11.00	12.00	71.79	51.28

*Indicates the referent

### Standardized change in CRC knowledge

Table 3 presents the results from the univariate logistic regressions for a standardized change in knowledge about CRC. The only statistically significant characteristics associated with a standardized change in knowledge about CRC were race (OR = 0.30; 95% CI: 0.11–0.80 for AA versus Whites) and intervention type (OR = 5.97; 95% CI: 1.94–18.43 for PowerPoint versus Inflatable Colon); that is, for example, for participants viewing the PowerPoint presentation, odds of a standardized change in knowledge of at least 0.35 were nearly six times that of those with an intervention type utilizing the Inflatable Colon. Univariate logistic regressions revealed no statistically significant changes in CRC knowledge scores based on the age, gender, education, CRC screening history, and family history of CRC.

**Table 3:** Frequencies and univariate logistic regressions for standardized change in knowledge about CRC among participants in the Screen to Save Initiative, April 2017 to September 2017

Characteristic		S_k_ $<$ 0.35 n (%)	S_k_ $\ge$ 0.35 n (%)	OR (95% CI)	p Value
Age	50–65^*^	24 (53.33)	21 (46.67)	1.00	0.33
	66–74	13 (41.94)	18 (58.06)	1.58 (0.63–3.98)	
Gender	Female^*^	26 (50.00)	26 (50.00)	1.00	0.75
	Male	12 (46.15)	14 (53.85)	1.17 (0.45–3.00)	
Race	White^*^	8 (29.63)	19 (70.37)	1.00	0.02
	AA	30 (58.8/2)	21 (41.18)	0.30 (0.11–0.80)	
Education	Not a college graduate^*^	24 (52.17)	22 (47.83)	1.00	0.64
	College graduate	14 (46.67)	16 (53.33)	1.25 (0.50–3.13)	
Insurance	Private only^*^	9 (40.91)	13 (59.09)	1.00	0.08
	Medicare only	13 (68.42)	6 (31.58)	0.32 (0.09–1.16)	
	Other	16 (43.24)	21 (56.76)	0.91 (0.31–2.65)	0.86
Screening history for CRC	Outside guidelines^*^	20 (54.05)	17 (45.95)	1.00	0.37
	Within guidelines	18 (43.90)	23 (56.10)	1.50 (0.62–3.67)	
Family history of CRC	No^*^	27 (50.00)	27 (50.00)	1.00	0.73
	Yes	11 (45.83)	13 (54.17)	1.18 (0.45–3.10)	
Intervention	Inflatable colon^*^	33 (61.11)	21 (38.89)	1.00	< 0.01
	PowerPoint	5 (20.83)	19 (79.17)	5.97 (1.94–18.43)	

*Indicates the referent

The multivariate logistic regression model for change in knowledge about CRC is displayed in Table 4. Race (OR = 0.23; 95% CI: 0.07–0.73 for AA versus Whites) and intervention type (OR = 6.19, 95% CI: 1.85–20.70 for PowerPoint versus Inflatable Colon) were the only statistically significant characteristics associated with a standardized change in knowledge about CRC in the model. Although insurance status was marginally statistically significant, it remained in the multivariate model primarily because it confounded associations between other factors (race and intervention type) and standardized change in knowledge.

**Table 4:** The multivariate logistic regression model for standardized change in knowledge about CRC among participants in the Screen to Save Initiative, April 2017 to September 2017

Characteristic		OR (95% CI)
Race	White^*^	1.00
	AA	0.23 (0.07–0.73)
Insurance	Private only^*^	1.00
	Medicare only	0.22 (0.05–1.00)
	Other	0.86 (0.26–2.90)
Intervention	Inflatable colon^*^	1.00
	PowerPoint	6.19 (1.85–20.70)

*Indicates the referent

### Intent to screen for CRC

The univariate logistic regressions for intent to screen for CRC are presented in Table 5. Education (OR = 0.42; 95% CI: 0.16–1.13 for college graduate versus not a college graduate) and any insurance coverage besides private insurance only or Medicare only (OR = 2.66, 95% CI 0.83–8.51 for other versus private only) were marginally statistically significant (*p* $\le$ 0.10). Univariate logistic regressions revealed no statistically significant changes in intent to screen for CRC based on age, gender, race, CRC screening history, family history of CRC, intervention type, and standardized change in knowledge score. In a multivariate model (not shown), after statistical adjustment for one another, no factors were significantly associated with intent to screen for CRC. A standardized change in knowledge about CRC (OR = 1.59; 95% CI: 0.58–4.36 for a standardized change in knowledge equal or greater than 0.35 versus a standardized change in knowledge less than 0.35) was not significantly associated with intent to screen for CRC.

**Table 5:** Frequencies and univariate logistic regressions for intent to screen for CRC among participants in the Screen to Save Initiative, April 2017 to September 2017

Characteristic		Do not strongly agree n (%)	Strongly agree n (%)	OR (95% CI)	p Value
Age	50–65^*^	12 (24.49)	75 (75.51)	1.00	0.84
	66–74	9 (26.47)	25 (73.53)	0.90 (0.33–2.4)	
Gender	Female^*^	18 (30.51)	41 (69.49)	1.00	0.15
	Male	4 (15.38)	22 (84.62)	2.42 (0.73–8.02)	
Race	White^*^	9 (32.14)	19 (67.86)	1.00	0.36
	AA	13 (22.81)	44 (77.19)	1.60 (0.59–4.38)	
Education	Not a college graduate^*^	9 (19.15)	38 (80.85)	1.00	0.09
	College graduate	13 (36.11)	23 (63.89)	0.42 (0.16–1.13)	
Insurance	Private only^*^	9 (37.50)	15 (62.50)	1.00	0.40
	Medicare only	6 (26.09)	17 (73.91)	1.70 (0.49–5.90)	
	Other	7 (18.42)	31 (81.58)	2.66 (0.83–8.51)	0.10
Screening history for CRC	Outside guidelines^*^	10 (25.64)	29 (74.36)	1.00	0.96
	Within guidelines	12 (26.09)	34 (73.91)	0.98 (0.37–2.59)	
Family history of CRC	No^*^	16 (26.67)	44 (73.33)	1.00	0.80
	Yes	6 (24.00)	19 (76.00)	1.15 (0.39–3.40)	
Intervention	Inflatable colon^*^	16 (28.57)	40 (71.43)	1.00	0.43
	PowerPoint	6 (20.69)	23 (79.31)	1.53 (0.53–4.47)	
Standardized change in knowledge	S_k_ $<$ 0.35^*^	12 (31.58)	26 (68.42)	1.00	0.37
	S_k_ $\ge$ 0.35	9 (22.50)	31 (77.50)	1.59 (0.58–4.36)	

*Indicates the referent

## Discussion

This study utilized two community-based educational interventions to increase CRC knowledge and intention to screen for AA and APP populations in Ohio. The interventions successfully increased CRC knowledge for participants. However, the findings revealed that a standardized change in knowledge was not associated with an intent to screen for CRC. Level of education attained was the only factor found to have a marginally statistically significant association with an intention to screen for CRC, with college graduates, counterintuitively, reporting a lower intention to screen. In contrast, a standardized change in knowledge about CRC was associated with race, intervention type, and insurance. Participants who participated in the Inflatable Colon tour and AA participants had lower standardized changes in knowledge. Insurance confounded associations between standardized change in knowledge and both race and intervention type.

It was surprising to discover that a standardized change in knowledge was not associated with an intent to screen because the results do not align with the predominant findings in the literature [35, 36]. However, a previous study using the Inflatable Colon discovered that a change in knowledge was not associated with an intent to screen [46]. The unique population studied offers several possible explanations to consider when interpreting the results. For instance, changes in knowledge may be less influential in altering screening behavior when baseline knowledge levels are high [50]. The median preintervention score of 11 out of 14 revealed that the participants entered the study well-versed on the basics of CRC. In addition, creating a behavioral change, such as obtaining CRC screening often requires knowledge, which educational interventions about CRC help promote. However, the change in knowledge may not be enough to drive the behavioral change [51]. For example, it is well known that a variety of barriers exist for CRC screening like income and health insurance [52]. Furthermore, the theory of planned behavior stipulates that intention can only be transformed into behavior if the individual has volitional control over the behavior [31, 32]. This lack of volitional control may explain why insurance status was marginally associated with intent to screen; it is uncertain whether responses pertained to attitudes towards the behavior or perceived behavioral control. Therefore, the gain in knowledge achieved may have been insufficient in light of the barriers.

Higher education levels are commonly associated with higher participation in CRC screening [53–55]. Therefore, it is counterintuitive that those with a college degree were less likely to report an intent to screen for CRC. Yet, a prior study found that more educated people report a lower intent to take preventative actions that decrease their risk for cancer as they perceive less need for cancer prevention since they have a greater access to routine medical care [56]. Also, rather than viewing those with higher education reporting less intent to screen for CRC, it may be more insightful to consider that those with lower education are reporting a strong intent to screen for CRC. The interventions provided may be more beneficial to those with lower education as compared to those with higher education.

In this study, race, intervention type, and insurance through Medicare only were independently associated with a standardized change in knowledge. Findings pertaining to race were similar to those previously reported [37, 46, 48]. The result that a standardized change in knowledge increased in a smaller proportion of AA compared to Whites may be due to differences in culturally appropriate messages delivered during the interventions. Consequently, steps, such as focus group testing should be taken to modify the content and delivery of future educational sessions so that all races experience similar changes in knowledge. The association between the intervention type and standardized change in knowledge is most likely due to multiple factors. Notably, the PowerPoint presentation and the Inflatable Colon tour were structured differently. PowerPoint session participants experienced approximately 50 min of educational instruction while the Inflatable Colon tour contained approximately 10–20 min of educational instruction, which may explain the greater standardized change in knowledge among the latter participants. Lastly, it is unclear why being insured through Medicare only confounded associations between the other factors and standardized change in CRC knowledge.

Neither intervention was associated with increasing intent to screen for CRC, but participants receiving the PowerPoint session had a greater standardized change in knowledge. However, it is important to consider the utility of each intervention for a variety of circumstances. For example, the Inflatable Colon is more attractive for drawing in community members with families to learn about CRC at community fairs than a 50-min PowerPoint session on the same topic. On the other hand, transporting the Inflatable Colon to a sufficiently large space and then assembling and disassembling it requires much more effort and real estate than hosting a PowerPoint session in a small classroom. Furthermore, while the PowerPoint session was associated with a greater standardized change in knowledge, both interventions increased the change in knowledge for the majority of participants; therefore, both interventions can be used as an effective teaching tool. The pros and cons of the various educational modalities should be strongly considered in the planning stage of future community-based interventions.

The impact of the interventions should not be diminished despite the finding that a standardized change in knowledge was not associated with intent to screen for CRC. The majority of participants gained knowledge about CRC from the interventions. Knowledge is a critical component that patients must possess in order to participate in shared decision making with their healthcare team. Therefore, promoting patient autonomy through education is imperative, whether the informed patient elects to undergo screening or not. Because vulnerable populations, such as AA and APP experience lower knowledge about CRC and screening, providing community interventions like the ones utilized in this study will help boost patient autonomy by enabling them to become more involved in their medical decisions with their healthcare providers [27, 58].

### Strengths and limitations

This study benefited from several strengths in its design. In specific, the interventions were aimed at helping AA and AP, which are vulnerable populations for CRC. In addition, the study was strengthened by examining standardized change in knowledge, rather than simply change in knowledge. Participants arrived to the study relatively knowledgeable, so there was little room for improvement for many participants. In contrast to change in knowledge, standardized change in knowledge better accounts for marginal gains made by high preintervention participants, although it does so at the expense of discounting marginal gains made by those with low preintervention scores. Overall, standardized change in knowledge measures how much participants were able to learn based on the possible remaining knowledge available to them, thus creating a more equitable metric to contextualize knowledge gains.

This study was not without limitations. The sample size was relatively small, participants were not randomized to receive an intervention (which would have the benefit of randomly distributing potentially confounding factors across interventions), and some additional potentially confounding demographic factors were not collected in this study. Also, the study focused on intent to screen rather than observing screening behavior; follow-up of participants to verify screening outcomes would provide important information about the relationships between knowledge gains and hard screening outcomes. In addition, there may have been some misclassification of CRC screening history for those who did not provide the date of their last screening. Furthermore, all participants were considered to have average risk of CRC, although more rigorous screening regimens are recommended for individuals with elevated risk factors for CRC [59]. Lastly, this study was designed to impact individuals, but the systemic factors previously discussed need to be targeted as well to mitigate the barriers influencing an individual’s intention to screen for CRC.

## Conclusion

This study focused on identifying factors associated with a standardized change in knowledge about CRC and intent to screen for CRC for AA and APP in Ohio. These findings may be useful in designing interventions to assist populations experiencing disparities in CRC across the United States. Knowledge about factors associated with change in CRC knowledge and intent to screen for CRC may help delineate potential future audiences that could benefit the most from these interventions. Furthermore, resources that facilitate screening may need to be offered in conjunction with a change in knowledge to modify screening behaviors since a change in knowledge alone is not sufficient to generate intent to screen. For example, in addition to utilizing the Inflatable Colon, providing onsite screening resources at health fairs, such as an FOBT/FIT, may be beneficial [61]. Future research should be conducted to determine how to best tailor the interventions provided in this study to maximize the end goal of improving CRC screening rates while still being aligned with the goals of the communities in which they serve.

## Data Availability

All data are available upon request.

## Code availability

Code is available upon request.
